# Production of Methane, Hydrogen and Ethanol from *Secale cereale* L. Straw Pretreated with Sulfuric Acid

**DOI:** 10.3390/molecules25041013

**Published:** 2020-02-24

**Authors:** Jarosław Domański, Olga Marchut-Mikołajczyk, Weronika Cieciura-Włoch, Piotr Patelski, Urszula Dziekońska-Kubczak, Bartłomiej Januszewicz, Bolin Zhang, Piotr Dziugan

**Affiliations:** 1 Department of Environmental Biotechnology, Faculty of Biotechnology and Food Sciences, Lodz University of Technology, 90-924 Lodz, Poland; weronika.cieciura-wloch@edu.p.lodz.pl (W.C.-W.); piotr.dziugan@p.lodz.pl (P.D.); 2 Institute of Molecular and Industrial Biotechnology, Faculty of Biotechnology and Food Sciences, Lodz University of Technology, 90-924 Lodz, Poland; olga.marchut-mikolajczyk@p.lodz.pl; 3 Institute of Fermentation Technology and Microbiology, Faculty of Biotechnology and Food Sciences, Lodz University of Technology, 90-924 Lodz, Poland; piotr.patelski@p.lodz.pl (P.P.); urszula.dziekonska-kubczak@p.lodz.pl (U.D.-K.); 4 Institute of Material Science and Engineering, Faculty of Mechanical Engineering, Lodz University of Technology, 90-924 Lodz, Poland; bartlomiej.januszewicz@p.lodz.pl; 5 College of Biological Science and Biotechnology, Beijing Forestry University, Beijing 100083, China; zhangbolin888@126.com

**Keywords:** *Secale cereale* L., rye straw, biofuel, methane, hydrogen, ethanol, chemical pretreatment

## Abstract

The study describes sulfuric acid pretreatment of straw from *Secale cereale* L. (rye straw) to evaluate the effect of acid concentration and treatment time on the efficiency of biofuel production. The highest ethanol yield occurred after the enzyme treatment at a dose of 15 filter paper unit (FPU) per gram of rye straw (subjected to chemical hydrolysis with 2% sulfuric acid (SA) at 121 °C for 1 h) during 120 h. Anaerobic digestion of rye straw treated with 10% SA at 121 °C during 1 h allowed to obtain 347.42 L methane/kg volatile solids (VS). Most hydrogen was released during dark fermentation of rye straw after pretreatment of 2% SA, 121 °C, 1 h and 1% SA, 121 °C, 2 h—131.99 and 134.71 L hydrogen/kg VS, respectively. If the rye straw produced in the European Union were processed into methane, hydrogen, ethanol, the annual electricity production in 2018 could reach 9.87 TWh (terawatt-hours), 1.16 TWh, and 0.60 TWh, respectively.

## 1. Introduction

At present, fossil fuels still contribute over half of the global energy demand. Consequently, this causes, *inter alia*, depletion of fossil fuel resources, greenhouse gas emissions, increased dust production, and other environmental pollutants. In response to the growing threat of global warming and greenhouse gas emissions, the use of second generation fuels is beginning to be a reasonable alternative to fossil fuels such as crude oil and coal [1]. Furthermore, the sources of fossil fuels are often economically or physically limited, therefore, searching for alternative sources of energy, free from these disadvantages is so important [2]. Renewable energy sources, such as biomass, wind, solar, hydroelectric provide an alternative to fossil fuels, however, in the case of biomass, the use of food crops for energy production is inadvisable because it reduces its access for people. Waste agriculture biomass, like for example straw, could be a good renewable energy source, however, nowadays this material is less frequently used by scientists for the production of biofuels like methane and hydrogen, compared to ethanol [3,4,5,6,7,8,9,10]. Bioethanol is mainly used as a fuel additive to reduce oil consumption. Also, the use of agricultural waste containing cellulose increases national energy independence, contributes to the improvement of the rural economy and reduces greenhouse gas emissions. The use of residues from the agricultural industry, such as straw, reduces greenhouse gas emissions by 60 to 94% compared to fossil fuels [11]. Obtaining fuel from biomass is a difficult task that requires pretreatment. Lignocellulosic biomass such as straw from *Secale cereale* L. (rye straw) represents a promising renewable bioresource for the production of chemicals and biofuels.

Lignocellulosic materials contain polymerized carbohydrates that must be liberated from the cellulose and hemicellulose by hydrolysis into simple sugars (glucose and other sugars) that can be easily fermented by microorganisms to produce form different chemicals, including biogas or ethanol. [9,12,13]. A major challenge of using plant biomass in biotechnology processes is how to convert the carbohydrates in the lignocellulosic feedstock into fermentable sugars efficiently and advantageously. The selection of a suitable pretreatment method can increase the yield of biogas or ethanol.

Acid hydrolysis is one of the most commonly used chemical treatment methods for lignocellulosic materials. In this method most often sulfuric acid is used at a concentration of 0.5–10% at elevated temperature (140–190 °C) and pressure. Moreover, the duration of the pretreatment usually does not exceed several minutes. The purpose of this type of treatment is lignin and hemicellulose degradation, which makes the lignocellulosic materials more susceptible for microorganisms in biotechnological processes. The advantage of the process is the virtually complete conversion of hemicellulose into simple sugars (e.g., xylose) and volatile fatty acids. In addition, sulfuric acid is relatively cheap. The disadvantages of this method include the possibility of creating inhibitors, e.g., furfural and its derivatives, during the process. The amount of inhibitors depends on the conditions of the process, e.g., the higher the acid concentration and the higher the temperature, the more inhibitors are formed. Furthermore, high acid concentrations require sufficiently corrosion-resistant reactors. Moreover, after the chemical pretreatment with sulfuric acid, methane produced in the anaerobic digestion process may be contaminated with hydrogen sulfide resulting from the transformation of sulfur compounds. However, the resulting furfural can be separated from the hydrolyzate and used as an intermediate in chemical synthesis [4,7,10].

Cultivation of cereals, that are used in agriculture all over the world, generates a large number of residues with potential applications in biotechnological processes. The most popular are rice straw (global production of 600 Mt), wheat straw (350 Mt), maize straw (max 377 Mt) and sugarcane bagasse (max 1045 Mt). The maximum achievable energy from the treatment of the abovementioned substrates is 108 exajoule (EJ) [7].

It is estimated that from 1 ton of wheat 0.5 to 2 tons of straw can be obtained (depending on the soil type and variety). Consequently, this gives from 4 to 16 million tons of rye straw, of which 40% is potentially used for non-agricultural purposes [14]. According to statistical data of the European Union, in 2016 about 7.68 million tons of rye (*Secale cereale* L.) have been collected from 2 million hectares of land. In 2017, farmers collected 7.65 million tons, while in 2018 it was 6.5 million tons and harvest forecasts for 2019 indicate an increase of around 25%. For example, the two largest rye producers in Europe, i.e., Germany and Poland, forecast an increase from 2.2 million tonnes collected in 2018 to 3.3 million in 2019 and from 2.3 million to 2.6 million [14]. The oldest method of straw utilization was and still is to use it as litter material. Rye straw has been subjected to the following pretreatments: ionic-liquid [9], ammonia [15], ozone [5,16] micromycetes [17], organosolv [18], liquid hot water [18], and steam explosion [18]. 

Smuga-Kogut and coworkers used the ionic liquid (1-ethyl-3-methylimidazolium acetate) as a substrate for the pretreatment of rye straw. During an 8 h process, at 120 °C, in a volume of 15 mL per 10 g of total solid, the authors obtained 22.45 g of reducing sugars per L. Next, they performed enzymatic hydrolysis using cellulase (300 mL) and cellobiose (200 mL) enzymes for 192 h at 45 °C and obtained 56.92 g of reducing sugars per L [9]. Perez-Cantu and coworkers used ethanol as the solvent for the organosolv process. The alcohol was used in an 8: 1 ratio and diluted with water in a 1: 1 ratio. The best variant of the experiment was carried out under the following conditions 190 °C, the addition of 1.3% H_2_SO_4_ and time of 1 h [18]. The conditions for the most efficient rye straw decomposition using the liquid hot water process were as follows: 200 °C, 10 min, flow 4 mL/ min and pressure 50 bar [18]. For steam explosion, the best lignin degradation yields were achieved at 200 °C in 10 min [18]. Varnaite and Raudoniene used *Galactomytes geotrichum* and *Myrothecium verruciaria* for degradation of the lignins in rye straw. After 30 days of enzymatic hydrolysis, they obtained a 47 and 40% reduction in lignins, respectively [17]. Domański and coworkers, studying the effect of ozonation on the pretreatment of rye straw before the anaerobic fermentation process, obtained an approx. 30% increase in methane yield [5]. When studying the pretreatment process of rye straw using at 60 °C and 90 °C, Domański and coworkers obtained the results which show that the concentration of VFA, COD and reducing sugars is increased. Compared to raw straw, pretreatment with 2% ammonia resulted in 5–24 times higher VFA concentration, 6–14 times more reducing sugars and three to nine times more COD [15].

However, reports considering the use of rye straw as a renewable material, including its pretreatment with acid are still scarce. The little interest in this substrate could have been caused by the fact that only a few countries have a high acreage of this grain. The only study on the distribution of rye straw using dilute sulfuric acid (up to 1.5%) was conducted by Sun and Cheng [10]. In this paper, changes in rye straw structure after the pretreatment by sulfuric acid as well as the potential possibility of the use of rye straw for obtaining substrates (for example monomer sugars) for biotechnological processes (e.g., anaerobic digestion, production of biofuels such as hydrogen and ethanol) have been shown. According to the best of authors’ knowledge, this is the first report concerning the use of the sulfuric acid for rye straw pretreatment for the production of methane, hydrogen and ethanol.

## 2. Results and Discussion

### 2.1. Products of Secale cereale L. Straw Pretreatment

Obtaining glucose, xylose, arabinose, formic and acetic acid, furfural and total and volatile solids, which can be successfully used as substrates in many biotechnological processes, can be facilitated thanks to chemical pretreatment of rye straw with sulfuric acid (Table 1). With increasing sulfuric acid concentration, total solid (TS) and volatile solid (VS) decrease from 16–22% for 1 h pretreatment and 28–33% for 2 h. However, for 10% sulfuric acid, these values increase by 7.7% and 10.5%, respectively. The highest glucose content (5.33 g/L) was observed for rye straw treated with 10% sulfuric acid for 2 h at 121 °C. Glucose content obtained after rye straw chemical hydrolysis (CH) was about 11.4 to 31.3 times higher compared with samples of rye straw treated with water for 1 h. What is more, the values were about 7.24 to 20.4 times higher for the 2 h process. The values of glucose were higher by 9 to 117% for a 2 h hydrolysis than for a 1 h process (Table 1). Guerra-Rodriguez and co-authors [19] studied the effect of sulfuric acid on rye straw and for 5% sulfuric acid at 130 °C and 3 h, whereby the glucose yield was 7.3 g/L. The authors found that glucose yield increases with increasing concentration of sulfuric acid, which is confirmed by the results of our investigation. The highest glucose yield was noted for the highest sulfuric acid concentration for 2 h. Sun and Cheng [10] described the degradation of rye straw with sulfuric acid (concentration of 0.5% to 1.5%) at 121 °C for 1 h. Their results revealed that glucose yield was constant and equaled approximately 35 mg per g of rye straw. The concentration of glucose released from cellulose depends on the concentration of the acid used for hydrolysis and the time of pretreatment. [10]. 

The results shown in Table 1 prove that the highest concentration of xylose (17.52 g/L) was observed in samples hydrolyzed with 2% sulfuric acid, for 2 h, and at 121 °C. The concentration of xylose obtained after rye straw chemical hydrolysis was from 6.74 to 8.15 times higher compared with samples of rye straw suffused with water for 1 h, while for 2 h process the values were from 3.7 to 7.1 times higher.

Results obtained by Guerra-Rodriguez and co-authors [19], showed that the highest concentration of xylose (19.7 g/L) was obtained for chemical hydrolysis of wheat straw with 1% sulfuric acid for 1 h at 130 °C. They observed an increase in the concentration of xylose with increasing temperature and acid concentration. This suggests that the decomposition of hemicelluloses, the main component of which is xylose, followed by the transformation of xylose to furfural and its derivatives occurred [19]. Similar results were observed in our work, where for the 1 h process the xylose yield increased with increasing concentration of sulfuric acid (up to 2% H_2_SO_4_) and then decreased by 17% in relation to the highest efficiency. For a 2 h process, this decrease was 48% of the maximum yield of xylose. Sun and Cheng [10] analyzing the degradation of rye straw with sulfuric acid (concentration of 0.5% to 1.5%) at 121 °C for 1 h found three times higher xylose concentration reaching 150 mg per gram of rye straw. Xylose and arabinose are the main products of hemicellulose decomposition [10]. 

The furfural content equaled from 0.003 to 1.656 g/L for a 1 h process and from 0.004 to 2.174 g/L for 2 h hydrolysis (Table 1). In both cases, the concentration of furfural increases with the concentration of sulfuric acid. The content of furfural correlates with the concentration of xylose and arabinose. With the increasing concentration of furfural, a decreasing content of arabinose and xylose were noted. This is related to the fact that furfural comes from the decomposition of xylose and arabinose. Similar conclusions were reached by Guerra-Rodriguez and others [19]. According to their research, the amount of furfural increases with the concentration of sulfuric acid and the time of hydrolysis. They achieved 4 g/L of furfural, using 5% of sulfuric acid, during 180 min process [19]. The higher the acid concentration, the more hemicellulose was broken down and more xylose was released into the solution. The more xylose was formed, the more it can transform into furfural under the influence of sulfuric acid [10]. Therefore, considering the use of rye straw hydrolysates as a carbon source in biotechnological processes, the pretreatment parameters should be selected in such a way that the concentration of furfural is as low as possible, as it has a negative effect on these processes [19].

The initial concentration of arabinose after thermal hydrolysis (TH) at 121 °C and time of 1 h is 0.02 g/L. It increases to 2.31 g/L during CH with 1% sulfuric acid and with increasing acid concentration its content in the hydrolysate decreases by 8.7% to 1.6%. A similar phenomenon was observed for the 2 h process, where the initial concentration is 0.14 g/L and then increased to 2.49 g/L and then decreased by 2% to 23.1% (Table 1). The decreasing concentration of arabinose with the increase of the concentration of sulfuric acid used in the study, suggests that the reaction of arabinose conversion to furfural, as in the case of xylose [19]. Guerra-Rodriguez and co-workers [19] achieved a maximum arabinose yield of 3.3 g/L for 1% sulfuric acid and 180 °C for 1 h for wheat straw. In contrast to these results, Sun and Cheng [10] investigating the decomposition of rye straw with the use of sulfuric acid at concentrations from 0.5% to 1.5% at 121 °C and time from 30 to 90 min, did not observe such a phenomenon. The concentration of arabinose ranged from 12 to 18 mg per g of straw. This is probably because the concentration of sulfuric acid was too low. The concentration of arabinose released depended only on the concentration of the acid used for hydrolysis and didn’t depend on the time of pretreatment [10].

The content of cellobiose in the hydrolysate increases from 0.027 g/L obtained by HT (121 °C for 1 h) to 0.815 g/L for 1% sulfuric acid hydrolysis. With the increasing concentration of sulfuric acid the content of cellobiose decrease to 0.144 g/L. For the 2 h process, the dependence is similar 0.034 g/L for the control, increase to 0.732 g/L for 1% sulfuric acid and decrease to 0.018 g/L for 10% sulfuric acid (Table 1). The decrease in cellobiose is probably related to its decomposition to glucose [20].

The concentration of acetic acid ranged from 0.23 g/L to 2.50 g/L for the 1 h process and from 0.21 g/L to 2.60 g/L for the 2 h process (Table 1). The observed increase in acetic acid concentration is correlated with the increase in the concentration of sulfuric acid used in acid hydrolysis. The results of the chemical hydrolysis of wheat straw with sulfuric acid obtained by Guerra-Rodriguez and co-workers [19] showed the concentration of acetic acid at the level of 2.3–2.6 g/L for 1% sulfuric acid and temperature of 180 °C. Acetic acid is derived from the hydrolysis of acetyl groups that are part of hemicellulose. Hydrolysates obtained from agricultural waste have a higher concentration of acetic acid. As the concentration of acetic acid increases, the probability of inhibiting the growth of microorganisms involved in biotechnology processes increases [19,21,22]. 

### 2.2. FTIR Analysis of rye Straw Before and After Chemical Hydrolysis

**Figure 1:**
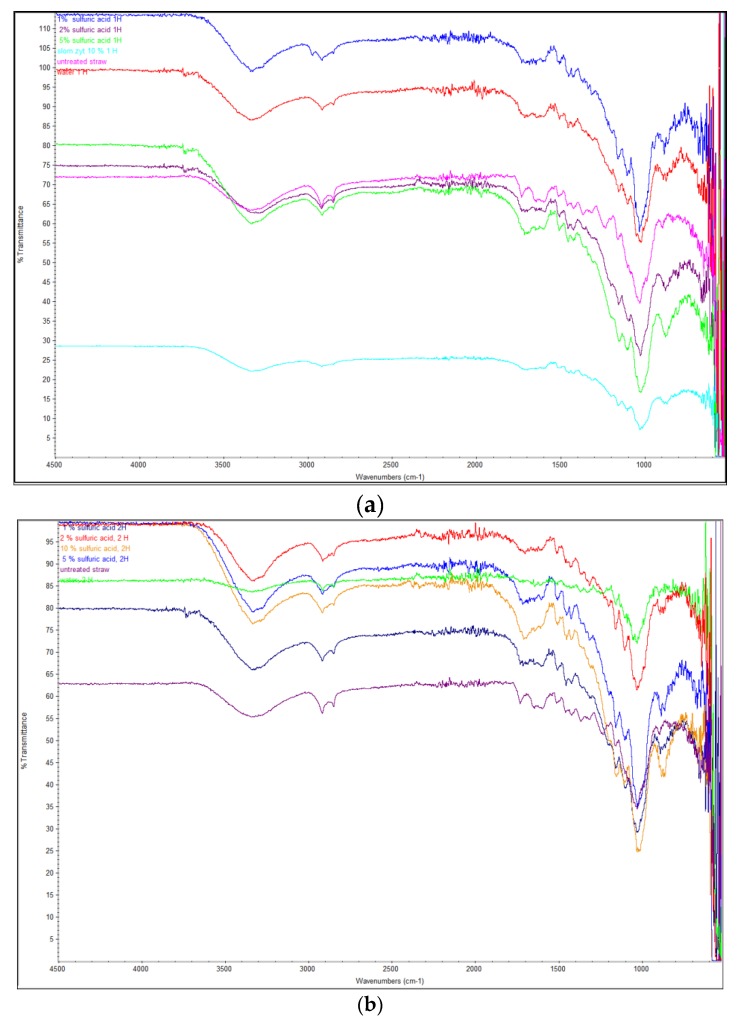
FTIR spectra of untreated and pretreated rye straw by different sulfuric acid concentration at 121 °C, 1 h (**a**) and at 121 °C, 2 h (**b**).

The FTIR spectra of rye straw hydrolyzed and non-hydrolyzed with sulfuric acid indicated changes in the chemical structure of pretreated biomass (Figure 1).

The raw rye straw showed strong hydrogen bond (OH) stretching at 3340.68 cm^−1^ and CH stretching of the aromatic methoxyl groups and methylene groups at 2918.19 cm^−1^. This band, related to phenolic and alcoholic hydroxyl groups is characteristic of all lignin IR spectra [5,23,24]. The same characteristics were noted for rye straw treated with sulfuric acid, although the transmittance in acid-treated samples was relatively lower, especially when sulfuric acid was used in 5% and 10% concentration. Grafted cellulose has a characteristic spectrum, which distinguishes it from the spectrum of the original cellulose. The transmittance for CH vibrations in cellulose and hemicellulose which occurred at 1367.19 cm^−1^ appeared at different intensities for the raw rye straw fibers and straw treated with water for 1 h at 121 °C. However, for variants pretreated with sulfuric acid, in all tested concentrations, the disappearance of these peaks was observed. A similar phenomenon was observed for the content of methoxyl groups in the lignin (1241–1233 cm^−1^), which remained the same for raw rye straw and straw after pretreatment with water for 1 h at 121 °C. Also, these vibrations were observed in the sample pretreated with 1% sulfuric acid for 1 h. In other variants of rye straw pretreated with sulfuric acid, and regardless of the acid concentration and time of pretreatment, the disappearance of these peaks was noted. COC vibrations in the anomeric region of hemicellulose occurred between 1162 and 1157 cm^−1^ [25]. In variants pretreated with sulfuric acid, a decrease in transmittance was observed, which suggests that the structure of hemicelluloses has changed. The lowest transmittance was observed for samples treated with 5% sulfuric acid, for 1 h. The content of CO stretching at C-3, CO stretching at C-6, and CC stretching, which occurred between 1048 and 1036 cm^−1^ was significant, with the lower transmittance in samples treated with 10 % sulfuric acid, both for 1 h and 2 h (24.09% and 24.8%, respectively) [26]. This proved that pretreatment with sulfuric acid makes cellulose easier to be degraded by microorganisms [5,26]. The same phenomenon was observed in the anomeric region (950–700 cm^−1^), and for peaks at 896 cm^−1^, which are indicative of β-glycosidic linkages between the sugar units and correspond to the C1 group frequency or ring frequency. A significant reduction in the transmittance was observed for all samples treated with sulfuric acid. However, the lowest transmittance for these vibrations was observed for ye straw treated with 10% sulfuric acid, both for 1 h and 2 h (47,4 and 41,8, respectively). This suggests the intermolecular degradation in the hemicellulose structure [5,27,28]. 

### 2.3. XRD Analysis

The values of the crystallinity index (CrI) for raw rye straw equaled 36.2%. After the chemical hydrolysis at temperature of 121 °C for 1 h the values were 60.9%, 61.1%, 52.1%, and 32.8%, respectively, for 1%, 2%, 5% and 10% sulphuric acid. On the other hand, under the same conditions, but a 2 h process, the index equaled 61.6%, 60.2%, 38.8%, and 21.56%, respectively, for the concentrations of the acid. Crystallinity index for rye straw subjected to a temperature of 121 °C (thermal hydrolysis) for 1 h process was 59.7%, while 56.7% was noted for 2 h process. In general, CrI is the ratio of crystalline to amorphous parts in the tested material. It is assumed that the amorphous parts, due to their disordered structure, are more susceptible to decomposition during pre-treatment. Cellulose contains crystalline and amorphous parts in its structure, while hemicellulose and lignin consist mainly of amorphous parts [29]. In our studies, an increase in CrI was observed with an increase in the concentration of sulfuric acid used for pre-processing of rye straw. The increase in the CrI is probably due to the distribution of lignin and hemicellulose, which consists mainly of amorphous parts, with a slight distribution of cellulose, which is made up of crystalline parts. These values were higher in comparison to the control sample (raw straw) as well as in comparison to samples of rye straw after HT. These results are in line with other studies [29,30,31]. However, increasing CrI was observed only for 1% and 2% of sulfuric acid. For 5% and 10% acid, the CrI values decreased. It may result, *inter alia*, from the destruction of crystalline structures of cellulose by sulfuric acid, increased temperature or extended process time. An additional argument is an increase in the content of glucose, which comes from the decomposition of cellulose. In the SEM image, destruction of the straw surface, as well as change in the colour of straw from yellow to black, were observed. This may indicate the physical destruction of the structure and, as a consequence, the decomposition of crystalline parts of straw. According to Zhao and co-workers [31], a decrease in the CrI values is caused by the swelling of cellulose [31].

### 2.4. SEM Analysis

**Figure 2:**
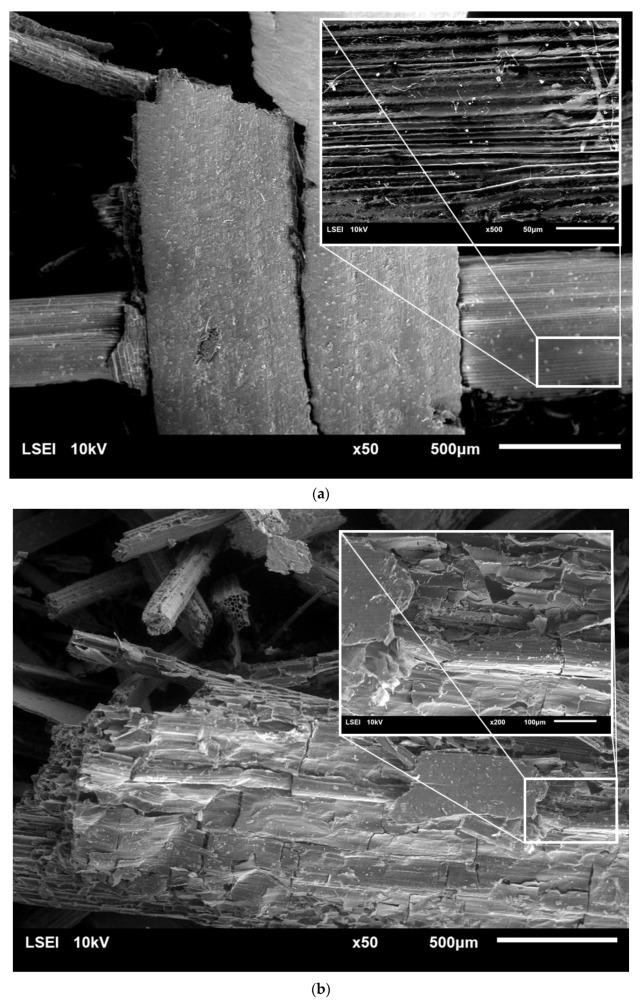

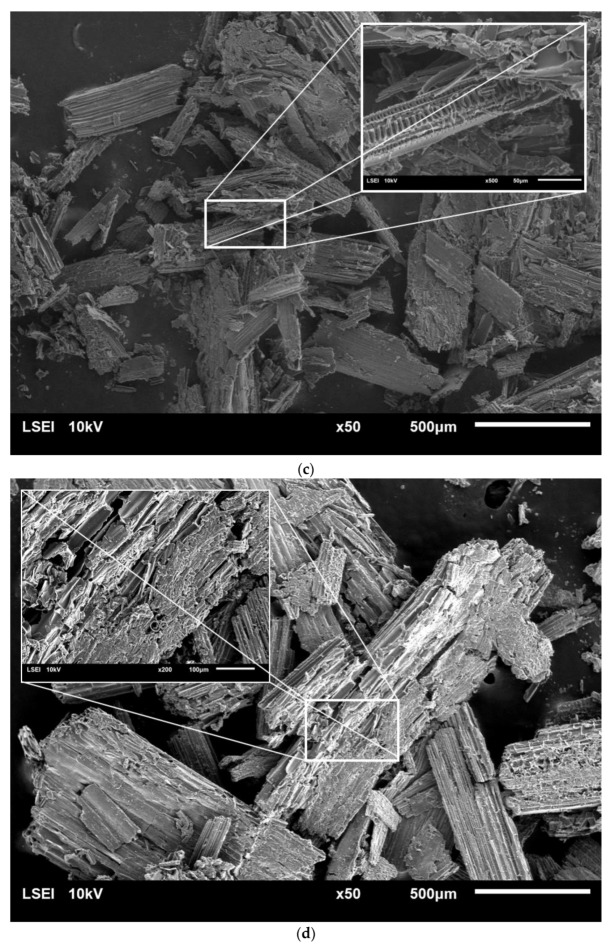

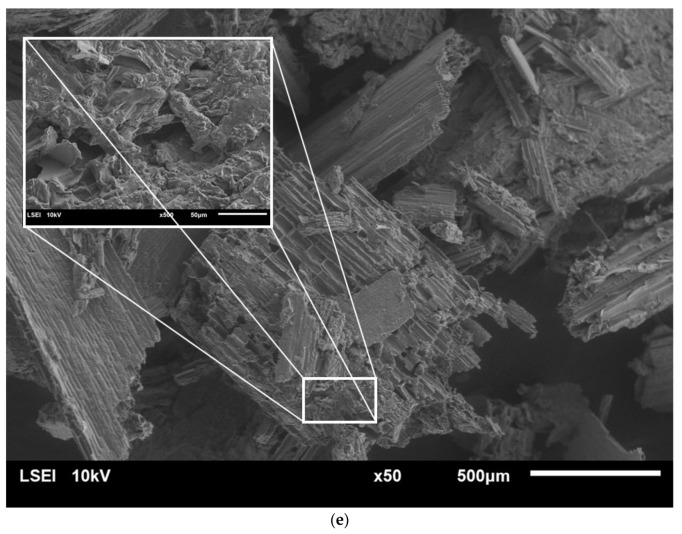
SEM images of raw rye straw under 50 and 200 or 500 magnification (**a**), rye straw after pretreatment with water at 121 °C and 1 h (**b**), rye straw after pretreatment with 5% sulfuric acid at 121 °C and 2 h (**c**) and 10% sulfuric acid at 121 °C and 1 h (**d**) and 10% sulfuric acid at 121 °C and 2 h (**e**).

Microscopic analysis using scanning electron microscopy (SEM) was carried out in to investigate the changes in the surface structure of raw rye straw, as well as of the straw after pretreatment (Figure 2).

Unprocessed rye straw was characterized by a smooth structure, composed of fine, ordered fibers arranged in parallel. Pretreatment with HT caused a rupture of the outer layer of rye straw. What is more, with the increase in the concentration of sulfuric acid and the time of the process, cracks in subsequent layers and fiber fragmentation were observed. Finally, the fibers’ structure was opened and its disorder increased. The more lignin and hemicellulose removed in the pre-treatment processes, was more open and exposed the straw structure was. What is more, porous structures were observed inside the straw fibers, which resulted in easier access to cellulose. Similar observations were noted by Zhang et al. [32] and Domański et al. [5]. Also, rye straw treated with 5% and 10% sulfuric acid turned black, which was also visible under the scanning microscope.

### 2.5. Ethanol Production

#### 2.5.1. Enzymatic Hydrolysis

The CH process carried out with different concentrations of sulfuric acid generated a low ethanol yield which did not exceed 1% of the theoretical yield. This was probably related to the lignin distribution. This matched the results obtained by Ponnusamya and co-workers [33], who described many lignin depolymerization products, including various kinds of alcohols [33]. On the other hand, the process of alcohol fermentation of rye straw hydrolysates after chemical hydrolysis, carried out with the use of yeast *Saccharomyces cerevisiae*, resulted in ethanol yields not exceeding 15% of the theoretical yield. Based on these results, it can be stated that the decomposition of rye straw using a thermal treatment of 121 °C and sulfuric acid is insufficient for the ethanol yield to be economically viable.

**Figure 3:**
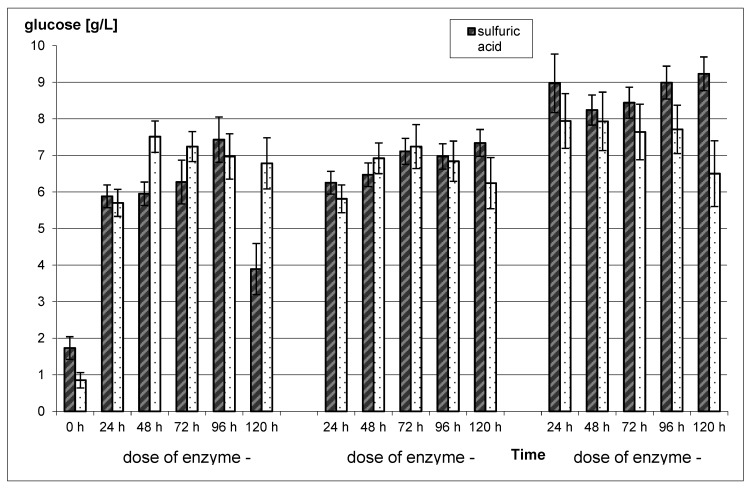
Enzymatic hydrolysis of pretreated rye straw using 2% sulfuric acid and water at 121 °C and 1 h. Control sample—Rye straw With water and sulfuric acid at 121 °C and 1 h—Not subjected to enzymatic treatment.

To increase the yield of ethanol and the economic viability of the process, enzymatic hydrolysis (EH) was carried out using a commercial enzyme, Novozyme Celic CTec2. As a pretreatment method, variant with the application of 2% sulfuric acid at 121 °C for 1 h was selected. Rye straw subjected only to heat treatment under the same conditions was used as a control. These conditions were chosen to limit the negative impact of formic acid, acetic acid and furfural as well as its derivatives on the ethanol biosynthesis. Both of these chemical compounds are very toxic for *S. cerevisiae*. Narendranath and co-authors [22] reported that 0.5% acetic acid exhibit an inhibitory effect on yeast growth [22]. Garay-Arroyo and co-authors [34] found that 2 g/L of furfural inhibits the reproduction of yeast cells [34]. In our study, enzymatic hydrolysis was carried out for 120 h in three doses of 5, 10, or 15 FPU per gram of rye straw. Samples subjected to analyses were collected every 24 h. The results after this process are shown in Figure 3.

Enzymatic hydrolysis of acid-treated rye straw resulted in an increase in glucose concentration by 225% to 534%, in comparison to non-hydrolyzed rye straw (Figure 1). The highest value of the examined parameter (9.24 g/L), was achieved for rye straw subjected to 120 h enzymatic hydrolysis with 15 FPU. Statistically significant differences (*p* < 0.05) between these three enzyme doses were noted. However, the differences between the obtained results within one dose were not statistically significant, except for the 5 FPU. This dose of the enzyme caused an increase in glucose concentration, from the beginning of the process to 96 h, and then its decrease by almost half (*p* < 0.05). In the case of rye straw subjected to thermal treatment and following enzymatic hydrolysis, glucose concentration, was from 671% to 934% higher compared to the control sample. However, glucose concentration for the control was half lower for the heat treatment only, compared to the acid treatment (*p* > 0.05).

Taking into account the increase in the glucose concentration (after acid pretreatment), the duration time of enzymatic hydrolysis is not important. This is due to the fact that its maximum concentration noted for 96 h for 5 FPU is only 26% higher in comparison to a 24 h process. What is more, a dose of 10 FPU the maximum is 17% higher for a 120 h process, while for 15 FPU a drop by 9% for 48 h, followed by an increase of 2% for 120 h was noted. For rye straw subjected to the heat treatment, EH caused an increase of the glucose concentration by 27% (5 FPU-48 h), 24% (10 FPU-72 h) and 12% (15 FPU-48 h) compared to the 24 h process. For 15 FPU dose, decreases from 72 h to 120 h by 4%, 3%, and 19%, respectively were observed.

Sun and Cheng [10] investigated the possibility of using rye straw for ethanol production. The authors applied 1% sulfuric acid at 121 °C and 1 h, and enzymatic hydrolysis with cellulase (25 FPU per gram of dry matter) and β-glucoidase (75 IU per gram of dry matter) within 48 h. They achieved 150 mg of glucose per gram of straw after the enzymatic hydrolysis process compared to the chemical hydrolysis itself-36 mg/g. Sun and Cheng [10] in their study achieved a glucose uptake of about 420%, which is similar to the result obtained in our study (an increase of 470% for 2% sulfuric acid and an enzyme dose of 15 FPU).

#### 2.5.2. Ethanol Fermentation

**Figure 4:**
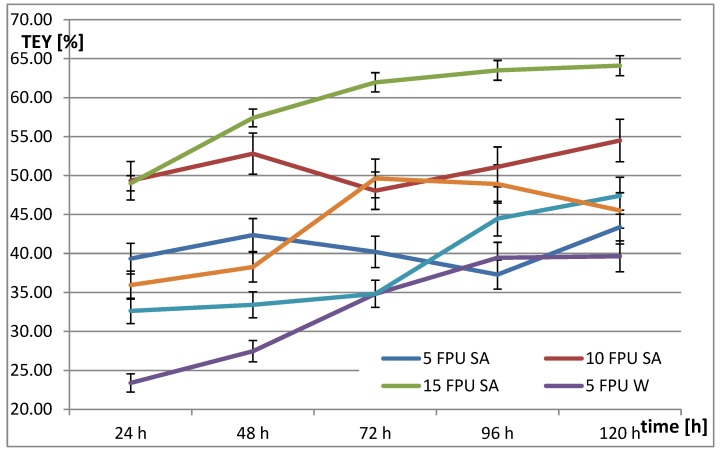
Theoretical ethanol yield (TEY) from rye straw treated with 2% sulfuric acid at 121 °C and 1 h after enzymatic hydrolysis (SA—Sulphuric acid, W—Water).

Ethanol fermentation (EF) of rye straw subjected to CH with 2% sulfuric acid at 121 °C for 1 h, allowed to obtain a theoretical ethanol yield of 13.25%. On the other hand, as a result of thermal hydrolysis (TH) at 121 °C and time 1 h, the theoretical ethanol yield equaled 22.06%. The use of EH resulted in the increase of the theoretical ethanol yield (Figure 4). For straw pretreated with sulfuric acid, the application of 5 FPU cased an increase from 116% to 320%, for 10 FPU from 363% to 411%, while for 15 FPU the values ranged from 370% to 484%. On the other hand, for rye straw with temperature pretreatment, the application of 5 FPU cased an increase from 106% to 180%, for 10 FPU from 148% to 251%, while for 15 FPU from 163% to 225%.

The highest theoretical ethanol yield was 64.10% for rye straw after CH combined with EH at a dose of 15 FPU after 120 h. For TH and EH, the highest theoretical ethanol yield was 46.63% for a dose of 15 FPU after 72 h. Ethanol fermentation of rye straw after CH and EH was 22.5% more efficient in comparison to TH and EH. However, this means that the ethanol fermentation process should last 48 h longer. For the same conditions (15 FPU and 72 h) CH and EH is more efficient by 19.9% from TH and EH, while for 15 FPU and 120 h, the obtained values were 29% higher, respectively.

The vast majority of research on the use of sulfuric acid for the pretreatment of various types of straw was carried out at high temperatures above 140 °C and low acid concentration, below 2%. However, Aditiya and co-workers [4] achieved 52.75% of the theoretical ethanol yield from rice straw pretreated with sulfuric acid (2 M, 90 min, 90 °C) and enzyme preparation (cellulase enzyme from *Trichoderma reesei* ATCC 26921 for 72 h). Robak and co-workers [35] obtained a 55% theoretical ethanol yield from pretreated rye straw with 1.5% sulfuric acid in the 1 h and 121 °C and 25 FPU of the commercial enzyme [35].

### 2.6. Batch Test 

#### 2.6.1. Methane Production

In order to establish the maximal biogas and methane yields from hydrolyzed rye straw, batch experiments were performed. Data of these trials are summarized in Table 2. The biogas and methane yields ranged from 248.15 to 594.6 L of biogas per kg of VS and 99.82 and 347.42 L of CH_4_/kg of VS, respectively. The highest methane yield of 347.42 L CH_4_/kg VS was obtained for rye straw hydrolyzed by 10% H_2_SO_4_ (121 °C and 1 h). This represents a 3.2-fold increase compared to the processing of rye straw only thermal treatment under the same conditions (121 °C and 1 h). Conducting the chemical hydrolysis process in 2 h gives worse results than in 1 h from 6.5% to 58.5% in the case of methane production. The methane yield increases with increasing sulfuric acid concentrations by 119, 127, 136, 224%, respectively, compared to the anaerobic fermentation of rye straw subjected to the action of temperature (121 °C) within 1 h of the process. Decreasing methane yields for 5% and 10% SA for a 2 h process can be caused by the formation of inhibitory compounds such as furfural, 5-HMF, etc. [36]. There are only a few articles on anaerobic digestion of rye straw; Peterson and co-workers [8] using wet oxidation has obtained an increase in methane by 34% for the best variant of the experiment. Similar results were shown by Domański and co-workers [5] using ozone as a reagent.

#### 2.6.2. Hydrogen Production

In order to establish the maximal hydrogen production from hydrolyzed rye straw, batch experiments were performed. Data of these trials are summarized in Table 2. The hydrogen yields ranged from 10.11 to 134.71 L of H_2_ per kg of VS. The highest hydrogen yield of 134.71 L was obtained for rye straw hydrolyzed by 1% H_2_SO_4_ (121 °C and 2 h). Similar results were obtained for 2% H_2_SO_4_, with a simultaneous shorter heat treatment time (121 °C and 1 h) and they were equal 131.99 L H_2_/kg VS. These values increased of 11 times compared to the processing of rye straw only by thermal treatment under the same conditions (121 °C and 1 h) and 13 times for two hours hydrolysis process (121 °C and 2 h).

The decreasing hydrogen yields produced by 5% and 10% SA for a 1 and 2 h process can be caused by the formation of inhibitory compounds such as furfural, 5-HMF, etc. Haroun and co-workers [36] showed that the increase in furfural concentration reduces hydrogen yield by up to 62% [36]. During the normal anaerobic digestion process, hydrogen also has been produced. Although the temporary yield of hydrogen reaches up to 12%, its total amount does not exceed 1% of the total biogas released. Only dark fermentation allows to change the end products of the anaerobic digestion process from methane to hydrogen [37].

### 2.7. Economic Evaluation of the Conversion of Straw From Secale Cereale L. to Biofuels 

An estimated analysis of energy yield from the conversion of rye straw (*Secale cereale* L.) biofuels such as methane, hydrogen, and ethanol was performed. The analysis of costs leading to the production of rye straw is difficult to achieve because it is reliant on the cultivation method as well as soil and climate conditions. The estimated cost of producing a kilogram of rye straw (VS) range from 0.014 to 0.166 euro [38].

The energy needed to grind rye straw is 0.044 megajoules (MJ) per kg VS, while thermal hydrolysis at 121 °C for 1 h and 2 h is 0.117 and 0.134 MJ per kg VS. Sulfuric acid should be diluted to obtain 1%, 2%, 5%, 10% concentration used for pretreatment of 1 kg of rye straw (VS). To this end, 0.12, 0.23, 0.58, 1.16 L concentrated sulfuric acid (95%) diluted with 10.89, 10.78, 10.43 and 9.85 L of water was used, respectively.

Cieciura-Włoch and co-workers [39] described that the energy obtainable from the combustion of hydrogen is 0.0107 MJ/L [39]. Our tests gave two largest similar amounts of hydrogen: 134.71 and 131.99 L H_2_/kg VS rye straw. This allowed obtaining a net energy in the amount of 1.309 and 1.279 MJ/kg VS, respectively. Głąb and co-workers [40] show that from one cubic diameter of methane an energy equal 0.036 MJ can be obtained [40]. Results obtained in the present work shown that for the best variant of treatment of rye straw with sulfuric acid net energy of 11.105 MJ/kg VS can be obtained.

The energy cost of converting biomass into ethanol is much higher than that of methane or hydrogen. This is due to the difficulty of converting all carbohydrates and lignocellulosic fractions into ethanol. First generation ethanol is processed from plant products that can be used by man in other ways. The energy needed for processes related to grinding corn maize and its mashing is up to 80% of the energy obtainable from bioethanol processing. The use of waste biomass, e.g., straw, reduces the cost of obtaining raw material and reduces energy demand through properly selected pre-treatment. The energy needed is only used for the fermentation and rectification of ethanol [40,41]. In our research, we received 0.04 L of ethanol from each kilogram of rye straw (VS), so the net energy gain was 0.674 MJ/kg VS. According to Głąb and co-workers [40] the calorific value of ethanol is 21.26 MJ per L [40].

The cost of cellulolytic enzymes used in pretreatment is difficult to estimate, as the price of the enzyme depends on the scale of ethanol production. Gomes and co-workers [42] reported that the cost of enzymatic treatment is between $ 0.10 and $ 0.68 per gallon of bioethanol. Thus, the cost of enzymatic treatment of 1 kg VS rye straw would be between 0.001 euro and 0.007 euro.

The production of methane from rye straw is the most profitable. Its yield is almost 8.5 times more profitable than hydrogen production and almost 16.5 times more than ethanol production. Assuming that 40% of rye straw produced is not used for agricultural purposes, in 2018 in the European Union an average of 2,600,000 megagram (Mg) remained (assuming from 1 Mg of rye we get 1 Mg of straw). Its pretreatment with sulfuric acid and processing in biotechnological processes to methane, hydrogen and ethanol would allow obtaining 9.87 TWh, 1.16 TWh and 0.60 TWh of energy, respectively. This amount corresponds to 8.2%, 0.9% and 0.4% of the annual electricity production in Poland.

## 3. Materials and Methods 

### 3.1. Substrate and Inoculum

Straw from *Secale cereale* L. was collected from a local farm in Lodz, Poland. The material was milled into pieces (4–12 mm) and stored in a grain bag made of polypropylene tape, at a 4 °C for further use. This was defined as untreated rye straw throughout this study. Chemical compositions of untreated rye straw was as follows: TS (total solid)—944.6 ± 2.4 [g/kg], VS (volatile solid) 908.0 ± 2.36 [g/kg]. Raw rye straw suffused with water contained: TS—220.9 ± 9.3 [g/kg], VS—214.35 ± 9.27 [g/kg]. Liquor after suffusion of rye straw with water contained (in g/L): glucose 0.07, xylose 0.09, arabinose 0.01, formic acid 0.05, and acetic acid 0.15.

Anaerobic sludge collected from an anaerobic mesophilic digester at the Municipal Wastewater Treatment Plant in Lodz, Poland and served as inoculum for batch experiments. The inoculum had total and volatile solids concentrations of 22.34 gTS/kg and 14.68 gVS/kg, respectively

### 3.2. Chemical Pretreatment

#### 3.2.1. Sulfuric Acid Pretreatment

The milled rye straw was pretreated with sulfuric acid (SA) (1%, 2%, 5%, 10 %) (*w*/*v*) in 250mL volume flask under the conditions of 121 °C (autoclaving) and S:L (solid: liquid) ratio of 1:10 (10 g rye straw in 100 mL SA in different concentration) for 1 h and 2 h, respectively. After the autoclaving process mixture was cooled to room temperature and slurry was separated by centrifugation (4000 rpm, 10 min) and supernatant was collected and stored at −18 °C for later analysis

#### 3.2.2. Sulfuric Acid Pretreatment for Alcoholic Fermentation

Milled rye straw in the amount of 300 g was placed in a glass container and filled with 3 L of 2% sulfuric acid. The prepared sample was heat treated at 121 °C for 1 h. To increase the bioethanol yield, the selected sample was subjected to an enzymatic pretreatment process. Therefore, in the next step, the sample was cooled to room temperature and the Cellic CTec2 (commercial enzyme preparation from Novozymes, (Novozymes A/S, Basgsværd, Denmark) was added at a dose of 5, 10, 15 FPU per gram of substrate. Samples were incubated at 50 °C for 120 h and samples were withdrawn every 24 h to analyze sugar monomers by HPLC. The pH samples before enzyme addition was adjusted to about 5 with 20% NaOH.

### 3.3. Batch Test

#### 3.3.1. For Methane Production

Batch experiments were designed to determine the biochemical potential of biogas and methane. Biomethane potential (BMP) was tested using a method described by Borowski and Kucner [43]. The water displaced method was used every day to measure the biogas yield. Methane was determined using a gas chromatograph (Agilent 7890A GC, Agilent Technologies, Santa Clara, CA, USA). 

#### 3.3.2. For Hydrogen Production

##### Preparation of Feedstock

To eliminate hydrogen consuming bacteria (primarily methanogens), the pH of feedstock was adjusted to 5.5 by addition 20% H_2_SO_4_. The experimental mixture was heated at 80 °C for 1.5 h, in a laboratory dryer. Also, the feedstock was subjected to thermal treatment processing [44]. Two experimental feedstock was prepared for each pretreated rye straw by sulfuric acid. One of them was used as control sample. In those samples, hydrogen consuming bacteria were not inactivated.

##### Fermentative Hydrogen Production

Biohydrogen production potential for each variant of the experiment (10 g rye straw, 100 mL sulfuric acid with different concentration—0%, 1%, 2%, 5% and 10%, 121 °C, 1 h and 2 h) was established using batch tests. For those variants 10 reactors were prepared. All batch tests were conducted in triplicates. The experiments were performed in 1000 mL glass bottles with a working volume of 700 mL. All batches were connected to the gas collecting tanks (volume–1 L). The measure of daily biogas production was counted by water displacement method. Hydrogen was measured using a portable gas analyzer (GA-21 plus, Madur, Zgierz, Poland). Five hundred g of inoculum were used to fill the reactors. In the next step the substrates were added to achieve the ratio of inoculum to substrate equal 2:1 based on volatile solids without any nutrients supplement [45]. To ensure anaerobic conditions the headspace of each bottle was rinsed for five minutes with nitrogen gas. In order to maintain constants optimum mesophilic temperature the bottles were incubated at 35 °C and their contents was shaken twice on day. The end of each experiment was established in point at which biogas production was observed. The dark fermentation trials ran maximally for 14 days.

### 3.4. Ethanol Fermentation

The fermentations were carried out in 200 mL flasks closed with rubber stopper and a fermentation tubes, each containing 50 mL of hydrolysate at 10% *w*/*v* solid loading. The hydrolysates were supplemented with (NH_4_)_2_HPO_4_ (0.3 g/L) and inoculated with dry distillers yeasts (Thermosacc Dry-Lallemand Ethanol Technology, Montreal, QC, Canada) at the dose of 0.5 g/L. Before the inoculation, the appropriate amount of yeast was suspended in warm water (approx. 35 °C) for 10 min to achieve better activity. The inoculated samples were incubated at 30 °C for 120 h under anaerobic conditions. After completing the fermentations, samples were analyzed for residual sugars and ethanol concentration by HPLC and GC methods respectively [46].

### 3.5. Characterization of Untreated and Pretreated Rye Straw

#### 3.5.1. Scanning Electron Micrograph (SEM)

To observe microstructural changes in rye straw treated or untreated with sulfuric acid caused by chemical pretreatment scanning electron microscopy (JSM-6610LV, JEOL, 3-1-2 Musashino, Akishima Tokyo, Japan) was performed, at an accelerating voltage of 30 kV. The rye straw sample subjected to the pre-treatment process was separated from the liquid and subjected to the drying process at room temperature for 48 h. The sample prepared in this way was used for imaging under SEM.

#### 3.5.2. Fourier Transform Infrared Spectroscopy (FTIR) Analysis

Infrared spectra of all rye straw samples—treated and non-treated with sulfuric acid were performed on a FTIR spectrophotometer (NICOLET 6700, Thermo Scientific, International Equipment Trading Ltd., Mundelein, IL, USA). For each measurement, spectra were obtained from 4000 to 400 cm^−1^ and 64 scans were collected. Equipment was cleaned between sample analysis using isopropanol and before each sample analysis background of the diamond window was subtracted [15].

#### 3.5.3. XRD Analysis

The crystallinity of treated straw samples was analyzed using X-ray diffraction (XRD). Each sample was ground to powder, placed in the sample holder and leveled off to obtain uniform X-ray exposure. Diffractograms were obtained using an Empyrean powder diffractometer (PANalytical, Empyrean, Malvern Panatytical, 7602 EA Almeo, Niderlandy). using CoKα radiation source of *λ* = 1.79 °A. A Goebel mirror together with 0.04 rad Soller slits and 1/2° divergence slit were mounted on a primary beam path. Diffracted intensities were recorded in continuous scan mode at room temperature over the angular range of 10° to 40°, under a step size of 0.1° and a time per step 4 s using Xe proportional counter with mounted 0.18° parallel plate collimator. The applied optics allowed avoiding any errors flatness and position connected. The crystallinity index (IC) was determined by using the Segal equation [47]:

*CI* = [(I(002) − I(am)/I(am)]∗100% (1)

where I(002) is the peak intensity counter reading at 2Θangle close to 26° representing crystalline material and I(am) is the counter reading at 2Θ angle close to 21° representing amorphous material in the samples.

### 3.6. Analytical Methods

Substrates were analyzed for total and volatile solids (TS, VS) and pH based on the Standard Methods for the Examination of Water and Wastewater [48]. A HPLC system (Agilent Technologies 1260 Infinity) was used to measure the concentrations of organic acids, sugars, furfural and ethanol in the supernatant. The process of separation of the compounds was conducted using a Hi-Plex h column (7.7 x 300 mm, 8 µm, Agilent Technologies). A refractive index detector (RID) was also part of this equipment. The temperatures of the column and detector were determined at the level of 60 °C and 55 °C, respectively. 0.05 M H_2_SO_4_ was used as a mobile phase (flow rate was equal to 0.7 mL/min). A volume of the analyzed sample was equal to 20 µL [22]. Ethanol yield (EY) was calculated according to the stoichiometric Gay-Lussac equation and expressed as a percentage of achievable theoretical yield [49]. The yield of ethanol after fermentation was calculated based on the equation as follows:

(2) $$\mathrm{Ethanol}\;\mathrm{yield}=\frac{\mathrm{Ethanol}\;\mathrm{concentration}\;\left[\frac{g}{L}\right]}{\mathrm{Glucose}\;\mathrm{in}\;\mathrm{hydrolysate}\;\left[\frac{g}{L}\right]\times 0.511}\times 100\%$$

An Agilent 7890A GC chromatograph with a TCD detector and a 2D column system (connected by a pneumatic switch): molecular sieve 5A, 60/80 mesh 6 ft × 1/8 in and Porapak Q 80/100 mesh, 6 ft × 1/8 in. was used to detection of methane concentration [50].

### 3.7. Statistical Analysis 

Analyses of all individual samples were carried out in quintuplicate. The calculation of mean values, standard deviations and the analysis of variance (single factor ANOVA) were performed using Statistica 10.0 (StatSoft, Kraków, Poland). The differences between individual means and control mean were tested using Tukey’s test. Significance was set at *p* = 0.05.

## 4. Conclusions

Results of the experiments show the usefulness of rye straw for biotechnological processes carried out to obtain clean energy, i.e., biomethane, biohydrogen and bioethanol. The most efficient form of pretreatment to obtain hydrogen is the use of 2% SA (121 °C, 1 h) and 1% SA (121 °C, 2 h). In the case of methane, the treatment with 10% SA at 121 °C for 1 h time is the most acceptable. The highest efficiency of ethanol production from rye straw was obtained after enzymatic hydrolysis preceded by an acid pretreatment (2% SA, 121 °C, 1 h) subjected to fermentation. Comparing the energy output from rye straw (*Secale cereale* L.), the most economically feasible seems to be the process of methane production by anaerobic digestion.

Although research on pretreatment of lignocellulosic materials is constantly evolving, there is still need for optimization of biotechnological processes related to biofuels production. The preliminary research of this study show the possibility of using rye straw as a raw material to obtain energy from renewable sources. However, to improve the efficiency of the results the studies should be continued under optimal conditions. Also, in case of anaerobic digestion process it is necessary to check the methane yield in the process of co-fermentation with other substrates, e.g., with liquid manure, which can be used to dilute the obtained rye straw hydrolyzate. In order to improve hydrogen yield, it would be necessary to optimize the dark fermentation process or try to perform it with the addition of other substrates that allow more efficient production of hydrogen.

## Figures and Tables

**Table 1:** Effect of rye straw pretreatment with different concentrations of sulfuric acid.

	TS [g/kg]	VS [g/kg]	Glucose [g/L]	Xylose [g/L]	Arabinose [g/L]	Cellobiose [g/L]	Formic Acid [g/L]	Acetic acid [g/L]	Furfural [g/L]
	**Pretreatment Temperature 121 °C Pretreatment Time 1 h**								
H_2_O	159.65 ± 5.32 ^a,b^	153.78 ± 1.84 ^a,b^	0.12 ± 0.00 ^a,b,c,d^	1.91 ± 0.41 ^a,b,c,d^	0.02 ± 0.00 ^a,b,c,d^	0.027 ± 0.001 ^a,b,c,d^	0.13 ± 0.004 ^a,b^	0.23 ± 0.01 ^a,b,c,d^	0.003 ± 0.000 ^a,b,c,d^
1% H_2_SO_4_	123.53 ± 0.63 ^c^	119.74 ± 1.43 ^a,c^	1.37 ± 0.02 ^a,f^	14.76 ± 0.59 ^a^	2.31 ± 0.01 ^a^	0.815 ± 0.017 ^a,e,f^	0.12 ± 0.007 ^c,d^	1.61 ± 0.06 ^a^	0.099 ± 0.006 ^a,e,f,g^
2% H_2_SO_4_	122.68 ± 3.32 ^a,d^	118.42 ± 6.53 ^b,d^	1.76 ± 0.04 ^b,g^	15.56 ± 0.43 ^b,e^	2.11 ± 0.08 ^b^	0.652 ± 0.021 ^b,g,h^	0.17 ± 0.004 ^e^	1.63 ± 0.08 ^b^	0.253 ± 0.012 ^b,e,h,i^
5% H_2_SO_4_	132.85 ± 9.17 ^e^	128.56 ± 8.89 ^e^	2.32 ± 0.09 ^c,e,h^	14.83 ± 0.61 ^c^	2.12 ± 0.03 ^c^	0.281 ± 0.009 ^c,e,g^	0.31 ± 0.011 ^a,c,f^	2.03 ± 0.11 ^c^	0.908 ± 0.051 ^c,f,h,j^
10% H_2_SO_4_	171.96 ± 7.09 ^b,c,d,e^	168.37 ± 6.81 ^c,d,e^	3.76 ± 0.11 ^d,f,g,h^	12.88 ± 1.01 ^d,e^	2.27 ± 0.07 ^d^	0.144 ± 0.011 ^d,f,h^	0.88 ± 0.012 ^b,d,e,f^	2.50 ± 0.16	1.655 ± 0.0814 ^d,g,i,j^
	**Pretreatment Temperature 121 °C Pretreatment Time 2 h**								
H_2_O	153.22± 11.73 ^a,b^	148.57 ± 11.48 ^a,b^	0.26 ± 0.01 ^a,b,c,d^	2.47 ± 0.32 ^a,b,c,d^	0.14 ± 0.01 ^a,b,c,d^	0.034 ± 0.003 ^a,b,c^	0.13 ± 0.002 ^a,b^	0.21 ± 0.02 ^a,b,c,d^	0.004 ± 0.000 ^a,b,c,d^
1% H_2_SO_4_	109.43± 8.9 ^c^	104.35 ± 8.58 ^c^	1.88 ± 0.02 ^a,e,f^	17.17 ± 1.12 ^a,e,f^	2.49 ± 0.26 ^a^	0.732 ± 0.029 ^a,d,e,f^	0.16 ± 0.006 ^c,d^	2.17 ± 0.11 ^a^	0.282 ± 0.012 ^a,e,f,g^
2% H_2_SO_4_	101.47± 2.79 ^a,d^	96.46 ± 3.11 ^a,d^	2.13 ± 0.06 ^b,g^	17.52 ± 1.34 ^b,g,h^	2.44 ± 1.81 ^b^	0.500 ± 0.037 ^b,d,g,h^	0.21 ± 0.005 ^e^	2.24 ± 0.09 ^b^	0.618 ± 0.033 ^b,e,h,i^
5% H_2_SO_4_	104.66 ± 0.28 ^b,e^	100.24 ± 0.43 ^b,e^	2.53 ± 0.11 ^c,h^	12.27 ± 0.87 ^c,e,g^	1.92 ± 0.98 ^c^	0.244 ± 0.014 ^c,gi^	0.44 ± 0.007 ^a,c^	2.27 ± 0.13 ^c^	1.066 ± 0.039 ^c,f,h,j^
10% H_2_SO_4_	169.24 ± 7.25 ^c,d,e^	164.06 ± 6.19 ^c,d,e^	5.33 ± 0.36 ^d,f,g,h^	9.04 ± 0.64 ^d,f,h^	1.92 ± 0.26 ^d^	0.018 ± 0.002 ^f,h,i^	1.16 ± 0.016 ^b,d,e^	2.61 ± 0.11 ^d^	2.174 ± 0.111 ^d,g,I,j^

The same lowercase letters in the table row indicate significantly statistical differences (*p* < 0.05) (under the same temperature and time conditions: 121 °C and 1 h or 121 °C and 2 h).

**Table 2:** Results and parameters estimated from batch digestion tests.

Parameter	Unit	10 g Rye Straw, 100 mL H_2_O	10 g Rye Straw, 100 mL 1% H_2_SO_4_	10 g Rye Straw, 100 mL 2% H_2_SO_4_	10 g Rye Straw, 100 mL 5% H_2_SO_4_	10 g Rye Straw, 100 mL 10% H_2_SO_4_
**Pretreatment Temperature 121** **°C** **Pretreatment Time 1 h**						
Mass of substrate	g	27.34	22.84	30.19	17.89	13.01
Substrate VS	g/kg	69.82	83.56	63.24	106.69	146.82
Mass of inoculum	g	500	500	500	500	500
Inoculum VS	g/kg	14.68 ± 0.15	14.68 ± 0.15	14.68 ± 0.15	14.68 ± 0.15	14.68 ± 0.15
SGP	dm^3^/kg VS	340.4 ± 18.5 ^a,b,c^	482.1 ± 3.7 ^d,e^	354. 9 ± 16.7 ^d,f,g^	437.5 ± 37.0 ^b,f,h^	594.6 ± 15.6 ^c,e,g,h^
SMP	dm^3^/kg VS	107.21 ± 21.1 ^a,b,c,d^	235.77 ± 8.9 ^a,e^	243.88 ± 19.7 ^b,f^	253.71 ± 21.8 ^c,g^	347.42 ± 16.1 ^d,e,f,g^
SHP	dm^3^/kg VS	10.11 ±1.4 ^a,b,c,d^	91.24 ± 4.7 ^a,e^	131.99 ± 5.7 ^b,e,f,g^	45.58 ± 4.6 ^c,d,f^	41.24 ± 7. 1^d,e,g^
		**Pretreatment Temperature 121** **°C** **Pretreatment Time 2 h**				
Mass of substrate	g	25.2	29.6	32.4	24.8	12.8
Substrate VS	g/kg	83.96	71.91	65.53	85.28	168.22
Mass of inoculum	g	500	500	500	500	500
Inoculum VS	g/kg	14.68 ± 0.15	14.68 ± 0.15	14.68 ± 0.15	14.68 ± 0.15	14.68 ± 0.15
SGP	dm^3^/kg VS	248.15 ± 10.2 ^a,b^	441.7 ± 23.1 ^a,c,d^	312.3 ± 41.6 ^c^	214.7 ± 31.8 ^d^	476.6 ± 47.8 ^b,c^
SMP	dm^3^/kg VS	99.82 ± 8.9 ^a,b,c^	189.94 ± 21.7 ^a,d^	155.99 ±16.8 ^b,c,f^	105.8 ± 11.4 ^d,f^	195.39 ± 21.4 ^c^
SHP	dm^3^/kg VS	12.15 ± 2.4 ^a,b,c,d^	134.71 ± 12.8 ^a,e,f^	105.81 ± 9.1 ^b,g,h^	60.53 ± 6.3 ^c,e,g^	48.51 ± 8.9 ^d,f,h^

SGP (Specific gas production); SMP (Specific methane production); SHP (Specific hydrogen production). The same lowercase letters in the table row indicate statistically significant differences (*p* < 0.05) (under the same temperature and time conditions of 121 °C and 1 h or 121 °C and 2 h).
